# Supplementary material for: Pre-Yield Non-Affine Fluctuations and A Hidden Critical Point in Strained Crystals
Source: Sci Rep. 2015 Jun 3;5:10644. doi: 10.1038/srep10644 (PMC4454149; doi:10.1038/srep10644)
Supplement: Supplementary Information [file srep10644-s1.pdf]

Supplementary Information for:

Pre-yield non-affine fluctuations and a hidden critical point in strained crystals

Tamoghna Das,[a,b] **Saswati Ganguly,**[b] **Surajit Sengupta**[*c] **and Madan Rao**[d‡]

[a] *Collective Interactions Unit, OIST Graduate University,1919-1 Tancha, Onna-son, Okinawa, Japan - 904-0495*
[b] *Centre for Advanced Materials, Indian Association for the Cultivation of Science, Jadavpur, Kolkata 700032, India*
[c] *TIFR Centre for Interdisciplinary Sciences, 21 Brundavan Colony, Narsingi, Hyderabad 500075, India.* email: surajit@tifrh.res.in
[d] *Raman Research Institute, C.V. Raman Avenue, Bangalore 560080, India*

1. The non-affine parameter χ, identification of non-affine clusters and Probability $P(\chi, \epsilon)$ of non-affine displacements as a function of strain

In this section, we give details of how we identify non-affine droplets in the 2D-LJ and 3D-LJ solids under pure shear [1]. Our procedure follows closely that used in Ref.[2]. Since all the particles in the ideal solid are identical, non-affine fluctuations in crystals arise solely from thermal effects. Non-affine regions therefore are composed of those particles which suffer somewhat large random deviations from their ideal lattice positions due to thermal motion. We identify particles in non-affine regions by computing the instantaneous non-affine parameter χ.

To obtain the non-affine parameter χ for a particle i we use a metric neighbourhood Ω around particle i defined using a cutoff size $\Lambda = 2.5$ corresponding to the range of interactions and minimise the error $\chi = \sum_j [\mathbf{r}_i - \mathbf{r}_j - (1 + \epsilon)(\mathbf{R}_i - \mathbf{R}_j)]^2$ with respect to choices of the strain tensor ϵ. Here $\mathbf{r}_i$ and $\mathbf{R}_i$ correspond to the instantaneous and reference positions of i, respectively and the sum is over the neighbours of i. A similar procedure was used in Ref.[3] to obtain a measure of non-affine displacements in amorphous solids. However, we modify the definition used in [3] in two important ways.

Firstly, we compute χ using a fixed reference $\{\mathbf{R_i}\}$. For the case where pure shear strain is zero (as in Ref.[2]) we have used the $T = 0$ ideal crystal lattice as reference. Where the solid is under pure shear, we have used as reference the ideal triangular lattice deformed by the uniform pure shear ϵ for our reference configuration. Secondly, the number of neighbours in Ω between the instantaneous and reference neighbourhoods is allowed to be different, accounting for *inclusions* within Ω which changes the metric coordination number. If there are no inclusions, the computed χ and ϵ are identical in meaning to that in [3]. If, however, there are inclusions, then extra contributions to both ϵ and χ arise from a change in coordination. In this case, the fitted ϵ looses its meaning as a local strain and

χ computes non-affineness in both displacements and the number of inclusions. For the parameters that we study, the number of inclusions is always at most one.

In Fig.S1 we plot the probability distribution $P(\chi)$ obtained for a solid of $\rho = 0.99$ for strains (pure shear) $0.0 \leq \epsilon \leq 0.072$. For a solid with fixed neighbors, $P(\chi)$ is an unimodal distribution[4]. When the number of neighbours is allowed to vary between the reference and the deformed configurations then $P(\chi)$ has additional peaks corresponding to each inclusion. As ϵ increases, the weight of $P(\chi)$ shifts to the peaks at large χ. From the distribution $P(\chi)$ we determine a cut-off $\chi_{\rm cut}$ above which particle displacements are defined to be *non-affine*. This is defined as in Ref.[2] by obtaining $P(\chi)$ for an equivalent harmonic system and equating $\chi_{\rm cut}$ with the value above which $P(\chi)$ for the harmonic lattice has negligible weight. This effectively discards 90% of the weight of the first peak in $P(\chi)$ (Fig.S1**a**) as coming from trivial harmonic fluctuations. All particles in a single snapshot having $\chi > \chi_{cut}$ are tagged and tagged particles residing within the first nearest neighbour shell of another tagged particle belong to the same cluster; any cluster having at least 7 particles is identified as a non-affine droplet.

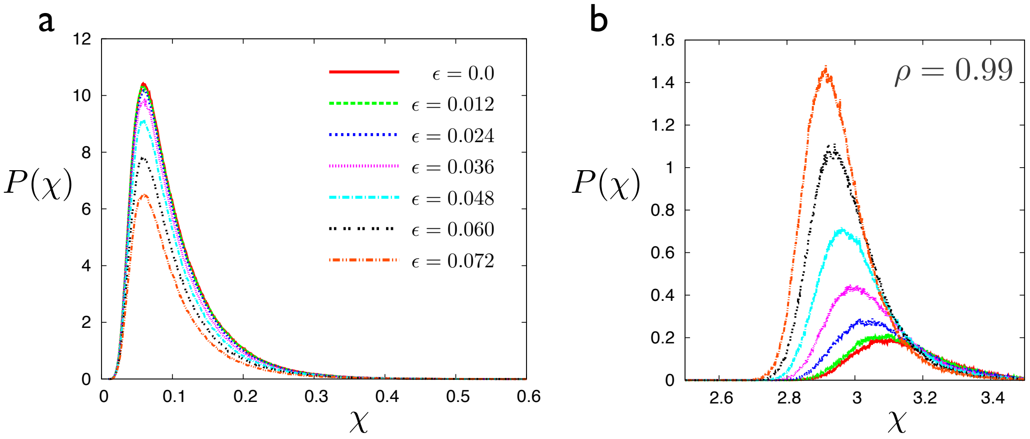

FIGURE S1. Normalized distribution of non-affine parameter $P(\chi)$ is shown for the case of $\rho = 0.99$ at $T = 0.4$. The two different figures **a** & **b**, shows two different ranges of the same distribution. Note the difference in the scale of the y-axis. $P(\chi)$ is essentially bimodal for the unstrained case as in Ref.[2]. Applied strain ϵ enhances non-affine displacements as observed by the increase in height of the second peak ($2.5 \leq \chi \leq 4.5$) at the expense of decreasing first peak ($\chi \leq 0.5$). Close observation to the local configurations reveals the fluctuations corresponding to the first peak keeps the numbers of neighbors intact within the cut off radius $\Lambda = 2.5$, the interaction range, the subsequent peaks arise when there are inclusions within the reference volume Λ changing the number of neighbors.

Our results are robust with respect to small variations of the cut-offs Λ and $\chi_{\rm cut}$. Increasing Λ increases the non-affine fraction while increasing $\chi_{\rm cut}$ decreases it. Large deviations of these numbers therefore shifts the focus from the fluctuations of interest namely all non-harmonic, non-affine fluctuations occurring within a neighbourhood of the dimensions of the interaction volume of the system.

A similar situation exists in 3D. We present the normalised distribution of χ as a 3D-LJ solid (face centred cubic) is strained via pure shear protocol (Fig.S2 (a) & (b)) as in 2D. In this case, we use a *metric* neighbourhood with $\Lambda = 1.1$ to obtain χ similar to the 2D-LJ case. As the system under consideration is deep in the solid region ($\rho = 1.5, T = 0.7$), the distribution $P(\chi)$ for the unstrained case ($\epsilon = 0.0$) is unimodal and breaks up into a bimodal distribution once sheared. First peak of the distribution decreases upon increasing shear at cost of higher, co-ordination number changing, non-affineness emerging in the system which contribute to the second peak. The situation is qualitatively in fair accord with its 2D counterpart. These distributions have been used to set the cut-off χ using the same criterion as in 2D and thus to define the non-affine clusters presented in the main text for the 3D case.

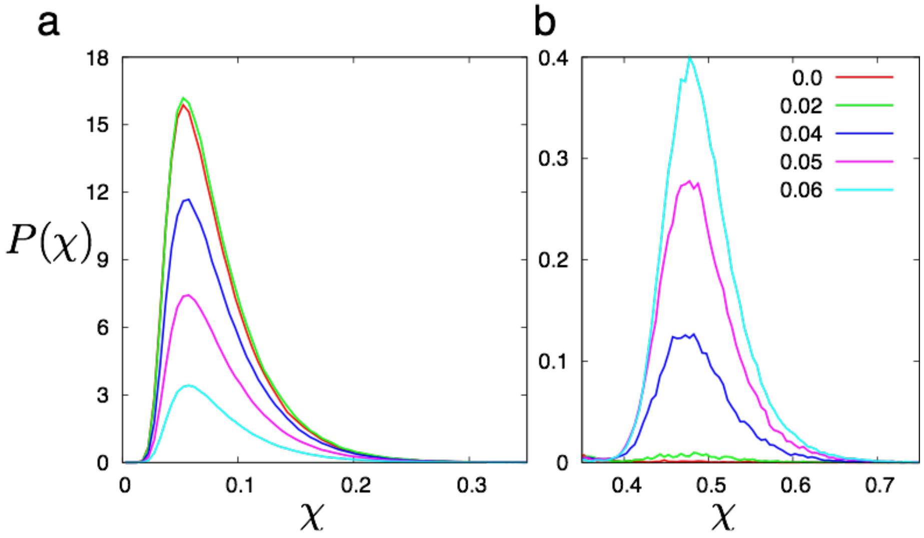

FIGURE S2. Increase in non-affineness upon shearing a 3D LJ solid (FCC) at $\rho = 1.5$ and $T = 0.7$ is shown in **a** & **b**. Both of them are part of the same distribution, but shown with separate axes for clarity.

2. Local thermodynamics of non-affine droplets in other model two dimensional solids

In the present paper [1] and our earlier work [2] we have related non-affine displacement fluctuations in the Lennard Jones (LJ) solid to droplet fluctuations from "nearby"

metastable states such as the liquid and glass. How general is this association and what are the limits of its validity ? To test the *generality* of this result, we carry out our analysis on another model solid where the particles interact with a different potential (for which it is known that both liquid and glassy free energy minima exist). To test the *limits* of this result, we study a model solid where, by construction, there are no locally stable phases except for the crystalline solid.

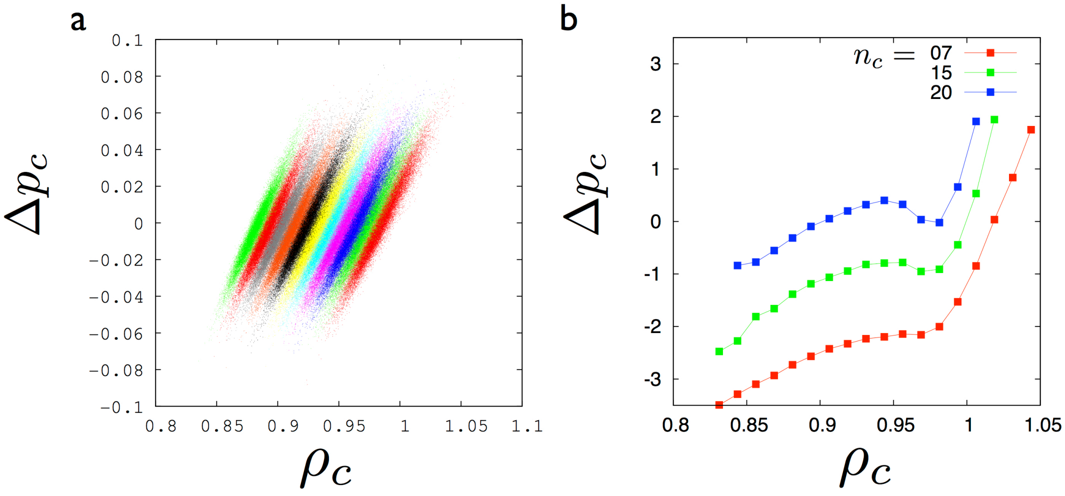

FIGURE S3. **a** A scatter plot showing the local pressure difference Δp_c as a function of the local density ρ_c for "non-affine" droplets in the harmonic triangular net obtained using a lower cut-off $\chi_{\rm cut} = 0.2$. The different colours correspond to solids at different densities obtained by applying a hydrostatic compression (or expansion) on the $T = 0$ solid with unit lattice parameter. The results show that in this system non-trivial droplet fluctuations do not exist and all fluctuations are normally distributed around the mean density and pressure. The slope of the plots correspond to the bulk modulus determined by the spring constant K which is the same for all the solids. **b** Plot of local thermodynamic quantities Δp_c vs. ρ_c for non-affine droplets in the WCA system at a $T^* = 0.4$ for droplets of size $n_c = 7, 15$ and 20. The prominent van der Waals loop in this quantity is similar to the corresponding plots (see Fig. 2 in [1] or Fig. 7(b) in [2]) for the LJ system.

2.1. **Harmonic triangular net.** The harmonic triangular net where particles in a two dimensional triangular lattice are connected to their nearest neighbors by harmonic springs (spring constant, K) is the simplest model of a *network* solid. The potential energy for

this system is given by,

$$V = \sum_{\langle ij \rangle} K(|\mathbf{r}_i - \mathbf{r}_j| - r_0)^2. \tag{1}$$

Here, $\mathbf{r}_i$ is the position vector of the $i-$th particle, r_0 is the equilibrium bond length and the sum extends over the nearest neighbor shell. Since defects, which change the connectivity of the lattice are not allowed by construction, such a solid never melts and the free energy surface in configuration space is particularly simple having just one quadratic minimum corresponding to the $T = 0$ ideal triangular lattice.

We have obtained the probability distribution of the non-affine parameter $P(\chi)$ in this system using the same procedure as used in the LJ system and using a neighborhood which includes the first two neighbor shells. The control parameter in our model is the lattice parameter $l_p = \sqrt{2/\sqrt{3}\rho}$ where ρ is the density of the solid and we hold the temperature T, the equilibrium bond length r_0 and K fixed at unity. The reference configuration is the ideal triangular lattice with the same lattice parameter. As in the LJ solid, the $P(\chi)$ is bimodal and resembles Fig.S1 and Fig. 2 in [2] for the unstrained solid at values of K low enough such that particles enter or leave the reference volume Ω. When non-affine droplets are defined using the same cutoff χ_{cut} as in [2], the local thermodynamics of these droplets as given by the Δp_c vs. ρ_c scatter plots are entirely unremarkable and are normally distributed around the average density of the solid. This is shown in Fig.S3 **a**. Further, this behaviour is, in this system, independent of the cut-off $\chi_{\rm cut}$ used. We do not see any evidence of a van der Waals loop in this case.

This is an important null result which shows that systems where there are no liquid like or glassy minima, do not have nontrivial local thermodynamics of the non-affine droplets and these droplets therefore do not correspond to fluctuations of any metastable minima.

2.2. **The Weeks-Chandler-Anderson solid.** In contrast to the harmonic solid, the system of particles interacting with the purely repulsive Weeks, Chandler and Anderson (WCA) potential [5] does undergo melting [6] and glass [7] transitions. This potential is closely related to the LJ potential and is given by,

$$\begin{aligned} V(r) &= 4(r^{-12} - r^{-6}) + 1 \quad r \leq 2^{1/6} \\ &= 0 \qquad\qquad\qquad\quad \text{otherwise} \end{aligned} \tag{2}$$

Unlike the LJ system, the WCA potential does not support a gas phase and there is no liquid - gas critical point.

We simulate a $N = 1024$ system of WCA particles in the NVT ensemble for several densities and a few temperatures. The configurations obtained are treated exactly in the same way as in the LJ solid and non-affine droplets are identified from the plots of $P(\chi)$. Local thermodynamics of these droplets then yield Δp_c vs. ρ_c curves which are qualitatively similar to the LJ case in [2].

We have thus shown that there is strong correlation between the appearance of non-trivial local thermodynamics of the non-affine droplets and the presence of metastable liquid or glass like phases. While not a complete proof, taken together, our results strongly

suggest that non-affine fluctuations in a crystalline solid are droplet fluctuations from nearby metastable minima.

References

[1] Accompanying manuscript T. Das, S. Ganguly, S. Sengupta and M. Rao.
[2] T. Das, S. Sengupta and M. Rao, Phys. Rev. E **82**, 041115 (2010).
[3] M. L. Falk and J. S. Langer, Phys. Rev. E **57**, 7192 (1998).
[4] S. Ganguly, S. Sengupta, P. Sollich, M. Rao, Phys. Rev. E **87**, 042801 (2013).
[5] J. D. Weeks, D. Chandler, and H. C. Andersen, J. Chem. Phys. **54**, 5237 (1971).
[6] A. Ahmed and R. J. Sadus, Phys. Rev. E. **80**, 061101 (2009).
[7] T. Kawasaki, T. Araki and H. Tanaka, Phys. Rev. Lett. **99**, 215701 (2007).
